# Crowdsourced Reconstruction of Cellular Networks to Serve Outdoor Positioning: Modeling, Validation and Analysis †

**DOI:** 10.3390/s23010352

**Published:** 2022-12-29

**Authors:** Andrea Brunello, Andrea Dalla Torre, Paolo Gallo, Donatella Gubiani, Angelo Montanari, Nicola Saccomanno

**Affiliations:** 1 Data Science and Automatic Verification Laboratory, University of Udine, 33100 Udine, Italy; 2 u-blox Italia SpA, Sgonico, 34010 Trieste, Italy

**Keywords:** spatio-temporal database, outdoor positioning, fingerprinting, crowdsourcing

## Abstract

Positioning via outdoor fingerprinting, which exploits the radio signals emitted by cellular towers, is fundamental in many applications. In most cases, the localization performance is affected by the availability of information about the emitters, such as their coverage. While several projects aim at collecting cellular network data via crowdsourcing observations, none focuses on information about the structure of the networks, which is paramount to correctly model their topology. The difficulty of such a modeling is exacerbated by the inherent differences among cellular technologies, the strong spatio-temporal nature of positioning, and the continuously evolving configuration of the networks. In this paper, we first show how to synthesize a detailed conceptual schema of cellular networks on the basis of the signal fingerprints collected by devices. We turned it into a logical one, and we exploited that to build a relational spatio-temporal database capable of supporting a crowdsourced collection of data. Next, we populated the database with heterogeneous cellular observations originating from multiple sources. In addition, we illustrate how the developed system allows us to properly deal with the evolution of the network configuration, e.g., by detecting cell renaming phenomena and by making it possible to correct inconsistent measurements coming from mobile devices, fostering positioning tasks. Finally, we provide a wide range of basic, spatial, and temporal analyses about the arrangement of the cellular network and its evolution over time, demonstrating how the developed system can be used to reconstruct and maintain a deep knowledge of the cellular network, possibly starting from crowdsourced information only.

## 1. Introduction

Our society is characterized in part by the pervasive use of mobile devices, which are successfully exploited in everyday life and in most business and industrial activities [1]. A distinctive feature of currently-used devices is that they encompass a variety of technological components, originally featured by dedicated, different pieces of hardware. The most significant example of such an integrated device is the smartphone, which offers a number of advanced and sophisticated services combining the traditional cellular communication system with other technological components, among which, typically, is a global navigation satellite system (GNSS) receiver [2]. IoT devices are another similar example.

Information about the position of a device is indeed exploited by a large array of applications, ranging from logistics and navigation to social activities and gaming. The global positioning system (GPS) is the most widely used GNSS, which allows devices to compute their location whenever there is an unobstructed line of sight to three or more satellites. Unfortunately, GNSS have some drawbacks. On the one hand, their signals are not always available. This is the case with environments such as indoor areas and urban canyons, where the performance of GNSS is significantly reduced, considering both the time required to obtain a position fix and the overall localization accuracy [3,4,5]. On the other hand, a GNSS may only be used sparely on battery-powered devices, due to its high energy consumption [6,7]. To overcome these limitations, hybrid positioning systems have been proposed, which pair GNSS with other localization technologies that can be used to replace or estimate the satellite-based position whenever necessary [8]. One of the most significant complementary/alternative solutions is offered by signal fingerprinting [9,10,11], where different signals received by a device are compared with those recorded in a radio map containing measurements taken at known locations to estimate the current position.

The cellular radio communication network, which appeared for the first time in the early 90s and then went through successive technological generations (GSM, UMTS, LTE, etc.), is nowadays the most widespread and used communication network, spanning the entire globe and allowing the transmission of both voice and data signals. The term cellular radio denotes the deployment of a large number of low powered cell towers for signal transmission; each one has a limited transmission area called a cell and is associated with a specific radio frequency. Given the global coverage of the cellular network and the common usage of its receivers, cellular signals are suitable for fingerprinting [12]. In such cases, a fingerprint consists of the collection of the signal strengths of the observed cell towers. It follows that they have a strong spatio-temporal characterization: the collected fingerprint is associated with a position (ground truth or estimated), and two fingerprints sensed in the same place may differ over time due to user equipment, environment, or cellular network changes.

Fingerprinting heavily relies on comprehensive and accurate knowledge of the cellular network configuration. Collecting such information is nowadays easy and convenient, thanks to the widespread use of mobile devices [13]. This led, over the years, to a very large amount of spatio-temporal data being collected via crowdsourcing and then stored in a variety of databases, some of which are open source. In this regard, the largest collaborative community project is OpenCellID (OpenCellID website: https://opencellid.org/, (accessed on 13 April 2022)), which, on average, collects more than 1 million new measurements per day. Unfortunately, data are not properly arranged: the repository contains lists of different values stored in tabular formats, e.g., csv, without any specific data structure reflecting the organization of the cellular network [14].

In this paper, we propose a general and flexible yet complete database schema for cellular networks that is modeled after the information available in signal fingerprints and capable of fostering the crowdsourced collection of data. As we will see, the system supports several operations, ranging from outdoor positioning to advanced spatio-temporal analyses and validation tasks pertaining to the state of the cellular network. Even though each generation of cellular networks is based on standard specifications, a major challenge is the fact that each operator adopts its own organization and makes some changes that are usually not known by the external people. As a result, the only way to get complete and accurate knowledge of the cellular network is to systematically collect and analyze available data. The task is exacerbated due to several characteristics of such a network being time-dependent, i.e., they undergo continuous changes. For instance, a cell may be created o removed, merged with another, or even spatially relocated [15]. Additionally, this continuous network evolution is taken into account and effectively managed by our proposed system, which overall demonstrates how a deep knowledge of the cellular network arrangement can be achieved and maintained based only on crowdsourced information.

To the best of our knowledge, no other comprehensive attempt to model and analyze the considered data has been reported in the literature. Previous works concentrated on limited network analyses, performed over specific technologies and with the purpose of solving precise problems, often relying on artificial intelligence techniques [16]. For instance, this has been the case with network optimization and planning tasks, such as the one in [17], where the authors propose an approach to determine groups of similarly behaving 3G cells, to support human experts in determining the state of the network; and the one in [18], where the collection of a large-scale dataset to foster mobile network planning is presented. In addition, a broad set of the literature focuses on anomaly detection and troubleshooting [19,20,21,22,23,24,25,26]. The only work close in spirit to ours lies in the indoor positioning domain [27]. The authors developed a framework based on a relational database that pairs heterogeneous, sparsely collected fingerprints with building topology information. The aim, besides that of supporting fingerprint-based positioning tasks, was to allow for the detection of spatio-temporal changes in the radio map and to provide a basis for advanced analyses.

This paper is organized as follows. Section 2 provides an account of the cellular network from the point of view of data modeling. It also presents the main issues related to the management of temporal aspects pertaining to the evolution of the network. Next, Section 3 presents the conceptual design of our original spatio-temporal database for the cellular network. The schema suitably models various generations of the network, independently of the data sources, and copes effectively with the previously described issues. The conceptual schema was translated into a logical relational schema and then physically implemented. Section 4 illustrates the process of network reconstruction and validation, by means of populating the database using two real-world crowdsourced datasets and introducing consistency checks that allow one to preserve the quality of the overall network configuration information. Section 5 provides a wide range of analyses based on SQL queries that show the capabilities of our system with respect to all the considered dimensions. Section 6 summarizes the work done and outlines future research directions.

## 2. An Overview of the Cellular Networks

Cellular networks, also referred to as mobile networks, can be viewed as the wireless extensions of traditional PSTN (Public Switched Telephone Network) and ISDN (Integrated Services Digital Network) systems. They support wireless communication between mobile devices for both voice and data transmissions, and allow for seamless nation or even worldwide roaming with the same mobile phone. Though in the past, cellular systems were developed by individual entities (companies, countries, etc.), today, cellular communications are based on standard definitions, and they span the entire globe. Different cellular technologies have been proposed over the years, each with its own peculiarities. A detailed account of the technical characteristics of wireless and cellular networks, such as the distinctive features of signals and antennas, can be found in many textbooks (see, for instance, [28,29,30]).

Our perspective here is different: we aim at providing a comprehensive conceptual model of cellular networks that captures all data relevant from the point of view of mobile devices; that is, rather than turning cellular network standards into a conceptual model, we build a representation that suitably integrates those elements that can be gathered by devices on the field (elements that, as we shall see, may not be contained in or differ from those actually declared in the standards). Such a model can then be exploited in several ways; for instance, it may help in answering individual positioning requests and in analyzing the behavior of large sets of devices (e.g., one may be interested in identifying recurring trajectories, or more generally, the spatial and temporal distributions of moving devices [31]). Furthermore, the model can be useful to check whether new observations are consistent with respect to the previously acquired information about the network, thereby allowing one to detect anomalies in the data and changes to the overall network arrangement.

In this section, to set the groundwork for the rest of the work, we describe the relevant architectural and administrative characteristics common to all existing cellular networks and the difficulties pertaining to their modeling. The interested reader may find the details of the specific mobile communication technologies (GSM, UMTS, LTE) in the Appendix A. For the purpose of clarity, in Table 1, we provide a list of technical acronyms that will be used throughout the paper.

### 2.1. General Features of Cellular Networks

The key notion in cellular networks is that of the cell—that is, the smallest division of the area served by a radio base station (coverage). By means of proper transmitting/receiving units, each base station generates one or more radio cell that allows the exchange of information among devices and their users.

#### 2.1.1. Architecture

**Figure 1:**
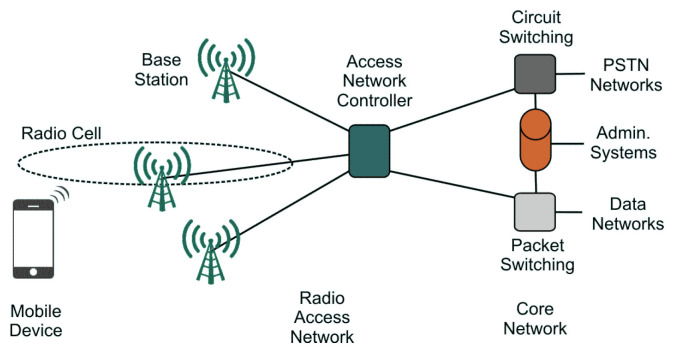
A general architecture for cellular networks.

Although the various technological solutions developed over the years differ from one another in several respects, they basically share the same network architecture, which conforms to the specifications of 3GPP [32] and is described, for instance, in [29]. It consists of three main levels: mobile device, radio access network (RAN), and core network (see Figure 1).

The mobile device level includes all the devices that use cellular services, such as phones, tablets, notebooks, and IoT appliances. Mobile devices are thus the part of the network which is directly managed by users. There are two types of device: a subscriber identity module (SIM), which contains information about users’ numbers and accounts, and mobile equipment (ME)—that is, any device capable of accessing cellular services.

The RAN level deals with the radio cells, which guarantee radio connectivity to the mobile devices. This level includes the base stations, which each control a set of cells and can be either omnidirectional (equipped with an antenna equally radiating in all directions) or sectoral (where each sector is generated by a different antenna). Sectored sites are the most common ones; there also exist mixed sites.

The core network level provides all the interconnection services that ensure voice, messaging, and data to be delivered to the required destination. In addition, it guarantees various security and administrative services. It supports circuit switching (CS), which is needed for real time connections, packet switching (PS), to manage services which do not require a prior reserved channel between endpoints for transmitting, and the administration layer, for billing, managing network user databases, and similar tasks.

#### 2.1.2. Cells and Their Administrative Organization

Cellular radio networks are based on the deployment of a large number of low-powered base stations for signal transmission, and each one has a limited transmission area called a cell. Each cell is characterized by a radio frequency and a geographical coverage area. Cells are grouped into clusters to avoid adjacent cells using the same frequency. Usually, a cell overlaps one or more others; a mobile device can distinguish among them by making use of their frequencies and scrambling codes (in the cases of UMTS and LTE).

The coverage of base stations may vary considerably, according to a straightforward hierarchical division, depending on their usage scenarios. As an example, in a rural environment, tall towers with large coverage areas are needed, whereas in urban canyons or indoor environments, smaller low-power antennas are exploited for better propagation of the signal, resulting in smaller coverage areas. Deploying a large number of small cells is also a commonly used strategy for dealing with a large number of users, as is typical for urban areas.

Cells in a mobile network are grouped together into administrative areas, known as location areas (LA) in 2G/3G voice services, routing areas (RA) in 2G/3G data services, and tracking areas (TA) in 4G networks. These administrative areas are used to determine in a rough way the current location of a mobile device in idle mode, that is, when it is switched on but is not using the network for any call or data exchanges. They play a fundamental role also in several other cases: when the mobile device switches to a different area and must be paged correctly; when it is switched on and a comparison with the previous identity is needed; and to perform periodic checks to ensure the network that the device is still reachable.

#### 2.1.3. Cell Identifiers

The cells of a network can be characterized by means of several identifying attributes at two different levels, which partially depend on the technology under consideration.

First of all, for each specific technological solution, there is a public land mobile network (PLMN), composed by two attributes:

- The mobile country code (MCC), which identifies the country where a network is located. It consists of 12 bits, or equivalently, three digits, and is assigned by the International Telecommunication Union (ITU). Some countries, e.g., USA and India, have more than one MCC;
- The mobile network code (MNC), which identifies a network in a country. It consists of 8–12 bits, or equivalently, 2–3 digits, and is assigned by the national authority.

The cell global identifier (CGI) is a unique global identifier that identifies each cell among all cells of all networks. It pairs the PLMN identifier with additional logical attributes that depend on the specific network generation. A mobile device can get these logical attributes by listening to information across the Broadcast Common Control Channel (BCCH). Since this operation can take some milliseconds, in order to avoid delays, networks provide other physical identifiers that make it possible to quickly discriminate cells without the need for decoding the BCCH. The physical-layer cell identifier provides quick discrimination between neighboring cells. It is a local identifier within a small part of an access network, and its definition depends on the specific technology.

### 2.2. Common Issues Related to the Management of the Networks

Irrespective of the kind of network (GSM, UMTS, LTE) considered, there are some underlying issues that have to be taken into account for successful modeling.

First, as previously remarked, reaching comprehensive knowledge of the network arrangement is of utmost importance to improve the localization performance of any cellular-network-based positioning system. Note how, in turn, this affects the effectiveness of any other task that exploits information regarding the positions of users, such as behavioral analysis, emergency responses, contact tracing, and logistics. Nevertheless, determining the state of the network arrangement is not trivial when relying on just crowdsourced measurements: often, the information sensed by the devices provides only a partial view of the environment—for instance, because they just aim to quickly discriminate between local cells. It is therefore, essential to make full use of the existing information, enhancing it through the already available network data and possibly exploiting related spatial knowledge.

Another major challenge is the inherent temporal evolution of the cellular network layout. Indeed, a number of reconfigurations can happen over time. For instance, in [15], the authors considered a cellular fingerprint dataset composed of 785,000 GSM observations collected in 2 years over a wide area of 1,800,000 km${}^{2}$, finding 198 instances of cells that had changed one or more of their identifiers. The phenomenon is named cell renaming—that is, a change, even a partial one, of the cell identifier (e.g., the LAC parameter for a group of cells). Observe that such an alteration has a temporal connotation, as it must hold that the first observation of a new cell takes place when the old one is not visible anymore. Moreover, the base station physically remains at the same place, having the same coverage, through the parameters of a cell change. Aside from cell renaming, base station relocations may be performed, where a cell gets assigned the same logical identifiers that were previously ascribed to a different one placed at another location. Such events are complex to model and deal with, as they are characterized by both a spatial and a temporal change. Moreover, renaming events concerning administrative areas (i.e., registration or routing areas) or the physical portions of the network (e.g., network controllers and base stations) are also to be hypothesized.

It is clear why these phenomena are a problem for localization if not properly handled. In the case of renaming, it would be impossible to exploit the information of the old cell, as it would not be associated with the currently visible one. This can naturally lead to a substantial degradation of the positioning performance in the affected area, as a considerable amount of data would suddenly no longer be available. The phenomenon would be accentuated in areas with a low cell density, such as rural regions. In [15], it is shown how accounting for such network changes allows one to increase the average number of candidates available for each position estimation by 12.1%. In the case of relocation, the problem would be an inconsistency of the data, which would again result in a positioning error, potentially of high magnitude, being closely related to the spatial displacement affecting the cell. Thus, a system that is able to effectively and efficiently manage the information required to identify these phenomena has great potential in the domain. Finally, observe that the actual identification of the previously mentioned phenomena (which can be delegated to appropriate algorithms) is only one side of the matter; the system needs also to be capable of harmonizing and keeping track of the old and the new data, to guarantee the overall consistency of the information before and after the network modification events.

## 3. Cellular Network Modeling

Dealing with cellular networks for positioning and other related tasks requires managing two different kinds of data: information on cellular networks and their spatio-temporal arrangement. In this section, we account for both of them by developing a conceptual model using a spatio-temporal extension of the classical entity-relationship notation called ChronoGeoGraph (CGG) [33,34].

Then, following consolidated database design methodologies, we translate the conceptual model into a relational logical schema, which has been implemented as a database within a PostgreSQL (PostgreSQL website: https://www.postgresql.org/, (accessed on 14 February 2022)) DBMS for evaluation purposes. As we will see, the design takes into account both spatial and temporal characteristics of the considered domain, in order to cope with the issues pointed out in Section 2.2.

The section is structured as follows. In Section 3.1, we consider the modeling of the network from a high-level perspective. Then, Section 3.2, Section 3.3, and Section 3.4 deal with the aspects that are specific to GSM, UMTS, and LTE technologies, respectively (although here we focus on GSM, UMTS, and LTE, the proposed model is highly flexible and modular with respect to network technologies). Section 3.5 combines all the previous local sub-models into a general, comprehensive one, which also includes the temporal aspects (detailed in Section 3.6). Next, in Section 3.7, the translation of the conceptual model into a logical relational schema that goes through a restructuring phase is illustrated. We conclude some remarks about the physical implementation of the database.

### 3.1. The Cellular Networks

**Figure 2:**
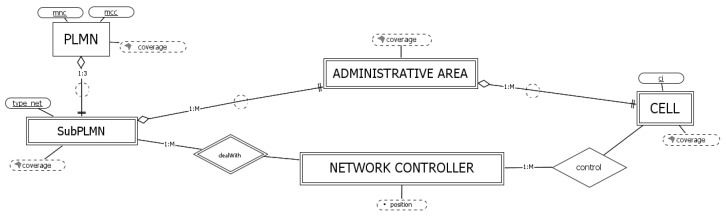
A high-level conceptual schema for cellular networks.

From a high-level perspective, regardless of the technology, cellular networks can be described with the entities depicted in Figure 2. Each network operator (entity PLMN) is identified by attributes *mcc* and *mnc* and is characterized by the derived spatial attribute *coverage*. Each PLMN is divided upon the basis of different cellular technologies (entity SubPLMN). Specifically, a PLMN is composed by 1 to 3 SubPLMNs (i.e., in the last case, the PLMN supports all three considered cellular technologies). Furthermore, the relationship between these two entities is a spatial aggregation: the attribute *coverage* of PLMN results from the combination of the coverages of the associated SubPLMNs. SubPLMN is a weak entity with respect to PLMN which has the attribute *type_net* as its partial identifier.

Considering the architectural structure of a network, each SubPLMN deals with one or more network controller, each managing, through some base stations, a set of cells. A network controller is a weak entity with respect to SubPLMN (as we shall see, the partial identifier, omitted here, depends on the specific network technology). Depending on the specific technology, the network controller can be incorporated into the base stations (as for LTE), or it can be organized into different components (as for GSM and UMTS).

From the administrative point of view, cells are grouped in administrative areas. Note the chain of spatial aggregations involving the attribute *coverage* of entities PLMN, SubPLMN, administrative area, and cell. It remains only to define how, practically, the calculation of such attributes is performed, an aspect that, together with the calculation of the network controller’s derived attribute *position*, is postponed to the introduction of the overall entity relationship diagram (Section 3.5).

### 3.2. GSM Networks

**Figure 3:**
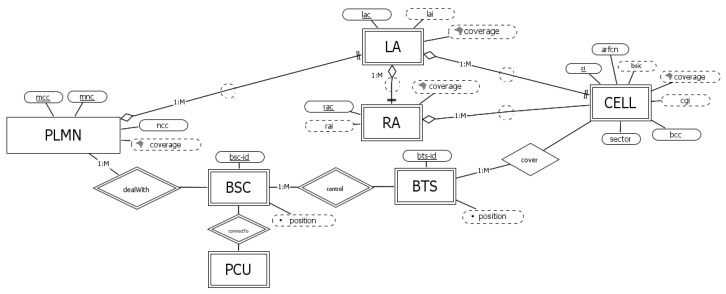
Conceptual schema for GSM networks.

As depicted in Figure 3, each GSM network operator (entity PLMN) is identified by attributes *mcc* and *mnc* and is characterized by the *ncc*.

As for the administrative part of the network, in GSM, administrative areas are divided into location areas (LAs) and routing areas (RAs); the former are uniquely identified within a PLMN by means of the corresponding code (attribute *lac*, to compose the *lai*), and the latter are identified within an LA by means of the attribute *rac*, or in other words, each RA is uniquely identified by the attribute *rai*, which is the combination of *lai* and *rac*. Each cell can be identified within its LA by means of its attribute *ci*. Note how in the diagram we chose this “administrative” way of identifying a cell, considering the positioning application and the standard applied by all operators.

Let us now turn to the physical unit of the network. A GSM PLMN deals with a set of base station controllers (BSCs), each of which is basically a switch in charge of frequency management among a set of base transceiver systems (BTSs), which are cellular towers whose radio signals cover one or more specific areas (entity cell). These components can be recognized through operator-defined attributes. For this reason, it is natural to specify a set of weak identifiers: the *bsc-id* identifying a BSC within its PLMN, the *bts-id* identifying a BTS within its BSC, and the *sector* identifying the cell within its BTS. In addition, at the physical level, each cell can also be locally characterized by *bsic* (that according to the standard definition is composed of *bcc* and *ncc*) and *arfcn*. Finally, note how each BSC is related to its packet control unit (PCU).

### 3.3. UMTS Networks

**Figure 4:**
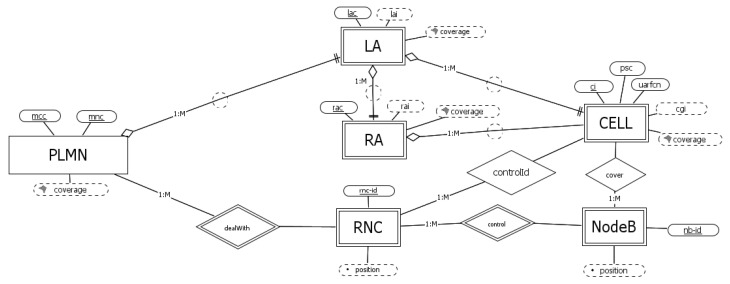
Conceptual schema for UMTS networks.

Other than the entity PLMN, UMTS networks have several differences with respect to GSM ones, as reported in Figure 4.

Administratively, considering the registration service, the cells are grouped in LAs, each identified by the *lai*, which is the combination of a PLMN identifier and *lac*. In a similar way, focusing on data routing, cells within an LA are grouped in RAs. Each RA is identified by the attribute *rai*, which is the combination of *lai* and *rac*. Each cell is globally identified by the *ci* within its LA (which is also the option we consider in the picture, for a more uniform description with GSM and LTE).

From the physical perspective, an UMTS PLMN deals with a set of radio network controllers (RNCs), weakly identified by *rnc-id*. Each RNC controls one or more base station (here called NodeB). Due to an absence of data about *NodeB* in positioning systems and a reference standard definition, we define it as a weak entity whose internal identifier (*nb-id*) can be operator-defined within the connected *RNC*. Each base station manages one or more *cell*. Finally, at the physical level, each cell can be locally identified by the combination of *psc* and the *uarfcn*. Note that the cycle given by the relationships among the entities *cell*, *NodeB*, and *RNC* is redundant. Nevertheless, we prefer to maintain it, since it gives us the added possibility to uniquely identify a *cell* with *ci* within its *RNC*.

### 3.4. LTE Networks

**Figure 5:**
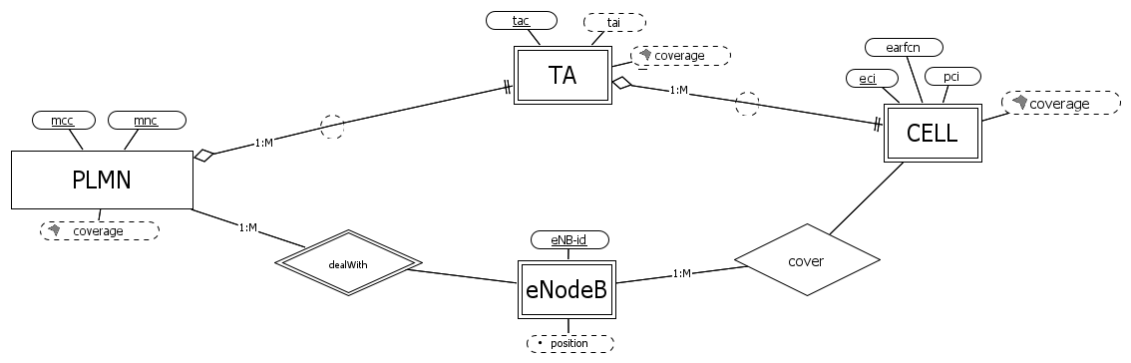
Conceptual schema for LTE networks.

The conceptual schema for LTE networks, depicted in Figure 5, shows its simplified organization.

Administratively, the cells are grouped in tracking areas (TAs), each one identified by its *tac* withing the PLMN. The combination of these identifiers is called *tai* (*mcc*+*mnc*+*tac*). The attribute *tai*, combined with the *eci*, globally identifies each cell (administrative identifier, chosen in the diagram).

Indeed, the radio access network (EUTRAN) includes only one component modeled by the entity eNodeB and identified by the attribute *eNB-id* within its PLMN. Each eNodeB controls one or more cell. Finally, considering the local physical identifier, for each LTE cell we have the composition of *pci* and *earfcn*.

### 3.5. The Overall Schema

**Figure 6:**
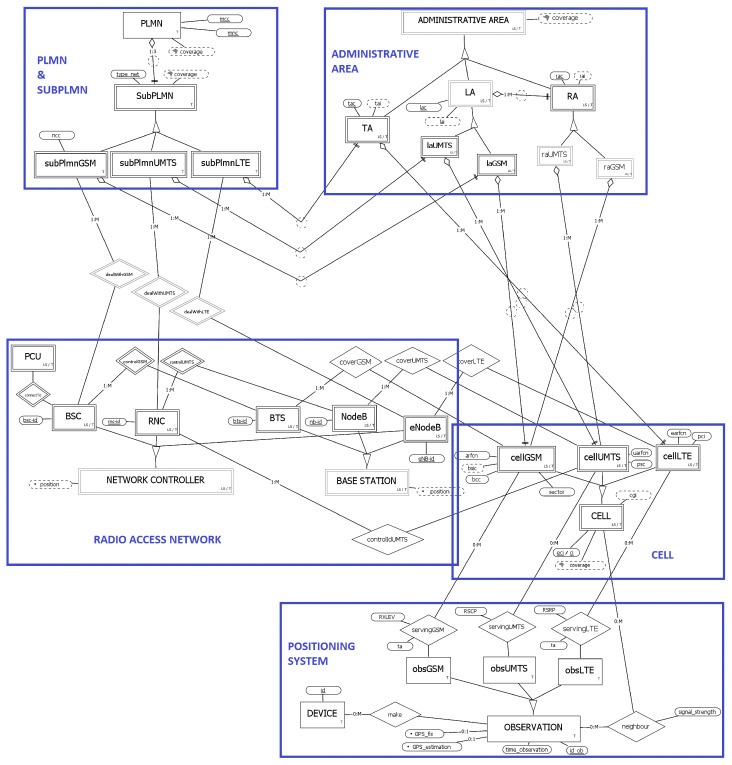
Complete cellular network schema, integrated with the part related to the positioning system and equipped with temporal support. T denotes entities that feature transaction time, whereas LS denotes instances that feature valid time.

We have now completed the illustration of all the components of the overall ER schema of the cellular network shown in Figure 6. The schema introduces several (total and disjoint) specializations that allow, for each entity, the modeling of the properties (attributes and relationships) that are specific to each type of network. As for the cell specialization, since we consider administrative identifiers for the cells, the partial key can be either *ci* (in the case of GSM or UMTS technologies) or *eci* (for LTE). In the schema, they are represented using the single attribute *eci/ci*.

The schema also includes the “POSITIONING SYSTEM” subschema, which represents the module in charge of dealing with the location of a device in space. In this work, we consider a fingerprint-based positioning system that makes use of cellular networks.

In the schema, a device (identified by its *id*) sends to the system several observations at different time instants. These observations can be associated with a GNSS position (*GNSSfix*) or not. If the GNSS position is not available, an estimation (*GNSSestimation*) may be derived using suitable positioning algorithms (dependently on the specific system and cellular technology; see [3] for fingerprinting techniques, especially signal-based ones). Observations sent to the system consist of data about one or more observed cell. One is the serving cell, to which the device is directly connected. The others are the remaining neighboring cells detected by the device. Each cell is received with a *signal_strength* that can have a different meaning according to the type of network under consideration: receiving level (RXLEV) for GSM, received signal code power (RSCP) for UMTS, and reference signal received power (RSRP) for LTE. Furthermore, for GSM and LTE serving cells, the timing advance (TA) could be detected, which measures the time a signal takes to reach the base station of the serving cell from the device (attribute *ta*). Of course, observations may in principle be extended with additional attributes encoding information such as the speed or the direction of a device, and the object that is being tracked (e.g., a person or an animal).

As mentioned in Section 3.1, it still remains to be explained how the *coverage* of a cell is calculated. This is done by aggregating the positions (attribute *GNSS_fix*) of the observations that have perceived the cell, either as serving or neighboring ones (e.g., calculating the convex hull).

The derived attribute *position*, which is part of the network controller and base station, can be inferred using the observations as well (there exist ad hoc algorithms; see, for instance, [35]). Moreover, from the position of the BASE STATIONs, one can then derive that of the linked network controller. Finally, the calculation of the remaining derived spatial attributes is trivial, since they can be obtained by aggregation of other spatial attributes.

### 3.6. The Temporal Aspects

A temporal database is a database that integrates support for handling data involving time. The temporal aspects of interest are the so-called valid time and transaction time: the first represents the time interval during which a fact is true in the real world, and it is a user-defined representation of time; the second represents the time interval during which a fact is current in the database, and it is a system-defined representation of time, namely, managed by the DBMS. A temporal database is physically implemented by augmenting the primary key of each table one wants to temporalize. Specifically, the key is extended with a pair of values for each temporal dimension, representing the extremes of the respective interval. A database that implements both valid and transaction time is referred to as a bi-temporal database.

The schema in Figure 6 reports the temporal aspects that have been associated with the entities, by means of the letters that are located on the bottom right corners of the corresponding boxes. Each entity of the cellular network has a transaction time interval (letter T). This allows us to easily retrace the database evolution, showing its content as it was at any previous moment in time. One could argue about the decision to maintain the observation’s *time_observation* attribute despite the presence of the transaction time; however, such values may be different, for example, due to a delay in the insertion of one instance into the database with respect to its moment of capture. Moreover, if updates are carried out on such database records (e.g., to correct errors), they might not have an impact on the *time_observation*, while certainly generating a new version of the record, tracked through the transaction time. In addition, considering that renaming may affect the cell, administrative area, and radio access network (as explained in Section 2.2), for each of the entities involved we also provide a valid time interval (identified by LS) that allows us to specify the moments in reality in which they existed. For example, this can be the case when we want to keep track of a cell that has been renamed and therefore no longer exists, but existed in a certain past time interval.

Finally, to make the distinction between transaction and valid time clearer, and to justify the presence of both of them, consider the following two scenarios (for the ease of reading, we adopt a simplified version of the tuples, not referring to the actual conceptual schema).

*Scenario 1.* On 2022-09-01, a new observation is added to the database, which, for the first time, reports the presence of a cell, say $cell_{x}$, which has never seen before. Thus, a tuple that describes the cell is inserted with both transaction and valid time intervals starting from 2022-09-01:

$$\begin{array}{c} \{\;\left(cell_{x},T:\left[2022\text{-}09\text{-}01,\infty \right],LS:\left[2022\text{-}09\text{-}01,\infty \right]\right)\;\}\;. \end{array}$$

The day after, a delayed observation, originally recorded on 2022-08-25, gets finally processed and inserted into the database. This observation also reports the presence of $cell_{x}$. As a result, the tuples that describe the event now are:

$$\begin{array}{c} \{(cell_{x},T:\left[2022\text{-}09\text{-}01,2022\text{-}09\text{-}02\right],LS:\left[2022\text{-}09\text{-}01,\infty \right]),\\ \;\;(cell_{x},T:\left[2022\text{-}09\text{-}02,\infty \right],LS:\left[2022\text{-}08\text{-}25,\infty \right])\;\}\;. \end{array}$$

Note how, in this way, the cell’s history is kept by means of the logically deleted tuple, while the current information is encoded by the newly added one.

*Scenario 2.* Valid and transaction times play a central role also in the management of a cell renaming or relocation. To reliably identify such an event, often multiple observations witnessing the change of parameters are needed, collected over an extended time frame (in general, the fewer the measurements related to a given area, the longer the period). Let us assume that a cell, named

$cell_{x}$, first detected and inserted in the database on 2017-07-12, changes part of its global identifier on 2022-09-01, thereby being detected from that moment on as a new cell, say $cell_{y}$. The situation is described by the following tuples:

$$\begin{array}{c} \{(cell_{x},T:\left[2017\text{-}07\text{-}12,\infty \right],LS:\left[2017\text{-}07\text{-}12,\infty \right]),\\ \;\;(cell_{y},T:\left[2022\text{-}09\text{-}01,\infty \right],LS:\left[2022\text{-}09\text{-}01,\infty \right])\;\}\;. \end{array}$$

Furthermore, assume that 10 measurements of $cell_{y}$ are needed to validate such information, and that we receive a new measurement each day. Thus, only on 2022-09-11 are we able to reliably detect the renaming event and merge $cell_{x}$ into $cell_{y}$. The situation now becomes:

$$\begin{array}{c} \{(cell_{x},T:\left[2017\text{-}07\text{-}12,2022\text{-}09\text{-}11\right],LS:\left[2017\text{-}07\text{-}12,\infty \right]),\\ \;\;(cell_{y},T:\left[2022\text{-}09\text{-}01,\infty \right],LS:\left[2022\text{-}09\text{-}01,\infty \right]),\\ \;\;(cell_{x},T:\left[2022\text{-}09\text{-}11,\infty \right],LS:\left[2017\text{-}07\text{-}12,2022\text{-}09\text{-}01\right])\;\}\;. \end{array}$$

Note that the “old” information pertaining to $cell_{x}$ has been logically deleted from the database using the transaction time on 2022-09-11, and a new tuple reflecting the more recent knowledge about it is added on the same date. Concerning the other information related to the two cells (e.g., their observations), they are dealt with in a similar way: after 2017-09-01, those of $cell_{x}$ become associated with $cell_{y}$ by means of changes to their validation and transaction times. This is particularly relevant from the point of view of positioning: when a new measurement detects $cell_{y}$, it can now leverage a larger set of observations than before the renaming operation.

### 3.7. Relational Database Development

In this section, we focus on the development of the relational database for crowdsourced cellular network reconstruction. In particular, Section 3.7.1 discusses its logical schema, which has mainly been derived from the entity-relationship diagram of Section 3.5. Although we do not describe here all the details regarding the physical implementation of the database, Section 3.7.2 still reports some notable aspects.

#### 3.7.1. Logical Schema

**Figure 7:**
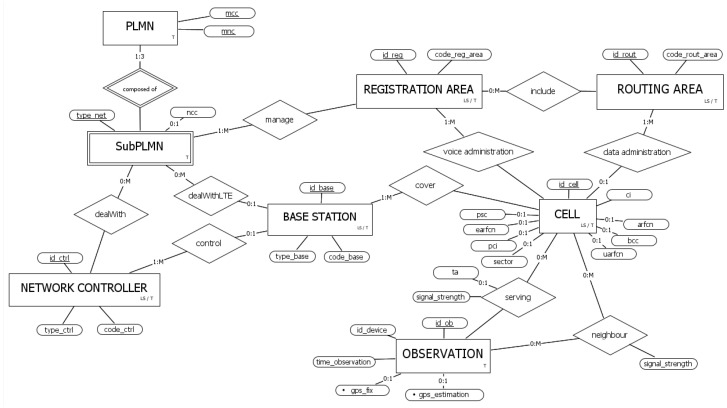
Restructured cellular network schema.

To translate the conceptual schema of Figure 6 into the logical one, preliminary restructuring of the former is needed, especially concerning the specialization constructs. In this restructuring step, we removed/combined unnecessary attributes, simplified entities, and introduced surrogate keys to effectively support the temporal capabilities of the system. The resulting restructured schema is shown in Figure 7.

First, the derived attributes are not reported in the restructured schema, as they will not be present in the tables of the database, given that it is possible to recover them through the definition of appropriate views. Specifically, the choice not to store *coverage* as a proper attribute is strongly dependent on the temporal dimension of the database: explicitly tracking its changes over time would result in an unnecessary overhead for the system, since the evolution of the coverage over time can be easily retrieved by appropriate temporal queries involving observation (precisely due to the lack of this spatial attribute, the aggregations of Figure 6 have been replaced here with standard relations).

Entities PCU and DEVICE have not been reported in the restructured schema, as they are of secondary importance, especially considering the purposes of the positioning system; to note their marginal roles, these entities have no attributes in Figure 6.

Let us now describe how the specializations were translated, omitting, for now, the roles played by the new primary keys that have been defined:

- Administrative area: two entities were introduced to deal with administrative areas: registration area and routing area.
  – The first entity focuses on the registration service (LA for GSM and UMTS, and TA for LTE); the attribute *code_reg_area* represents *lac* for 2G/3G and *tac* for 4G.
  – The second entity deals with the routing service, where differently from the first one (only for RA in GSM and UMTS), the attribute *code_rout_area* represents *rac*.
- For all the following specializations, it was decided to keep just the parent entity:
  – Network controller: the attribute *code_ctrl* groups the partial identifiers of the (former) children and *type_ctrl* denotes the type of controller (based on the considered cellular technology).
  – Base station: the attribute *code_base* groups the partial identifiers of the (former) children and *type_base* indicates the type of base station (based on the considered cellular technology).
  – SubPLMN: the optional attribute *ncc* represents the homonymous value considered in GSM technology only.
  – Cell: all the physical-layer cell identifiers values of different technologies are now modeled as optional attributes in the parent entity. Moreover, without loss of generality, a single attribute *ci* is used to represent both *ci* and *eci*.
  – Observation: the attribute *id_device* actually replaces the entity device.

As for the primary keys depicted in the restructured schema, they were defined as follows. Since the children of base station specialization had, in the previous schema, identifying relationships with different entities (precisely, BTS and NodeB with the network controller, and eNodeB with SubPLMN), to keep the parent entity by eliminating the children, the artificial attribute *id_base* was introduced as the new primary key. Note how the relations towards network controller and SubPLMN are now optional as a result of this choice. Finally, entities with a valid time interval are now strong entities identified by a serial key: registration area is identified by *id_reg*, routing area by *id_rout*, cell by *id_cell*, and network controller by *id_ctrl*.

The reason for the introduction of such surrogate keys is twofold. First, consider the possible renaming operations involving those entities, which can affect attributes that are part of their primary keys (remember that, in a bi-temporal database, the primary key of a temporalized entity is augmented with the attributes representing the transaction and valid time intervals, allowing the system to keep a history of each instance composed of all its subsequent modifications). Let us assume, for example, a renaming operation affecting a given cell $cell_{ci1}$ (where ci1 denotes the value of its attribute *ci*), turning it into $cell_{ci2}$. As changing part of the primary key means having a new, independent entry, the aforementioned event would result in not being able to recognize that $cell_{ci1}$ evolved in $cell_{ci2}$, as no link would be present among the corresponding (temporal) records, both still present in the database. Introducing a surrogate primary key id_cell solves the issue, as it is going to be immutable after the first appearance of the cell. In the considered case of renaming, only the attribute *ci* would change; thus, it would still be possible to recognize that the cell with id_cell=1,ci=1, evolved in id_cell=1,ci=2, linking the information between older and newer versions of the same cell. Note that such a procedure applies to any scenario where we merge multiple cells together: *ci* can be used to retrieve all the records in cell composing the actual cell (within a given administrative area), and *id_cell* discriminates between the single (merged) entries. Recalling the renaming example from Section 3.6, we now have the following scenario:

$$\begin{array}{c} \{(id\_cell=1,ci=1,T:[2017\text{-}07\text{-}12,\infty ],LS:[2017\text{-}07\text{-}12,\infty ]),\\ \;\;(id\_cell=2,ci=2,T:[2022\text{-}09\text{-}01,\infty ],LS:[2022\text{-}09\text{-}01,\infty ])\;\}\;\\ \;\;\;\;\;\;\;\;↓\mathrm{rename}\;ci=1\;\mathrm{in}\;ci=2\;\mathrm{on}\;2022\text{-}09\text{-}11\\ \{(id\_cell=1,ci=1,T:[2017\text{-}07\text{-}12,2022\text{-}09\text{-}11],LS:[2017\text{-}07\text{-}12,\infty ]),\\ \;\;(id\_cell=2,ci=2,T:[2022\text{-}09\text{-}01,\infty ],LS:[2022\text{-}09\text{-}01,\infty ]),\\ \;\;(id\_cell=1,ci=2,T:[2022\text{-}09\text{-}11,\infty ],LS:[2017\text{-}07\text{-}12,2022\text{-}09\text{-}01])\;\}\;. \end{array}$$

Compared to the case where *ci* is part of the (temporal) primary key and the surrogate key does not exits, here it is possible to retain the fact that (id_cell=1,ci=1) evolved in (id_cell=1,ci=2). Note that, after the renaming, the cell with ci=2 is logically composed of two records, and that cell ci=1 is not alive by itself anymore. Of course, the procedure can be repeated indefinitely, always preserving the entire knowledge about network’s reconfigurations:

$$\begin{array}{c} \{(id\_cell=1,ci=1,T:[2017\text{-}07\text{-}12,2022\text{-}09\text{-}11],LS:[2017\text{-}07\text{-}12,\infty ]),\\ \;\;(id\_cell=2,ci=2,T:[2022\text{-}09\text{-}01,\infty ],LS:[2022\text{-}09\text{-}01,\infty ]),\\ \;\;(id\_cell=1,ci=2,T:[2022\text{-}09\text{-}11,\infty ],LS:[2017\text{-}07\text{-}12,2022\text{-}09\text{-}01]),\\ \;\;(id\_cell=3,ci=3,T:[2022\text{-}09\text{-}15,\infty ],LS:[2022\text{-}09\text{-}15,\infty ])\;\}\;.\\ \;\;\;\;\;\;\;\;↓\mathrm{rename}\;ci=2\;\mathrm{in}\;ci=3\;\mathrm{on}\;2022\text{-}09\text{-}20\\ \{(id\_cell=1,ci=1,T:[2017\text{-}07\text{-}12,2022\text{-}09\text{-}11],LS:[2017\text{-}07\text{-}12,\infty ]),\\ \;\;(id\_cell=2,ci=2,T:[2022\text{-}09\text{-}01,2022\text{-}09\text{-}20],LS:[2022\text{-}09\text{-}01,\infty ]),\\ \;\;(id\_cell=1,ci=2,T:[2022\text{-}09\text{-}11,2022\text{-}09\text{-}20],LS:[2017\text{-}07\text{-}12,2022\text{-}09\text{-}01]),\\ \;\;(id\_cell=3,ci=3,T:[2022\text{-}09\text{-}15,\infty ],LS:[2022\text{-}09\text{-}15,\infty ]),\\ \;\;(id\_cell=2,ci=3,T:[2022\text{-}09\text{-}20,\infty ],LS:[2022\text{-}09\text{-}01,2022\text{-}09\text{-}15]),\\ \;\;(id\_cell=1,ci=3,T:[2022\text{-}09\text{-}20,\infty ],LS:[2017\text{-}07\text{-}12,2022\text{-}09\text{-}01])\;\}\;. \end{array}$$

The second reason pertains to the propagation of updates in the database. If, for instance, a natural key attribute of a given registration area ra1 is changed, in the schema of Figure 6, this would have caused a cascading update of all the foreign keys of cells connected to ra1. Again, this unnecessary computational burden is avoided with the introduction of a (never changing) surrogate key.

**Figure 8:**
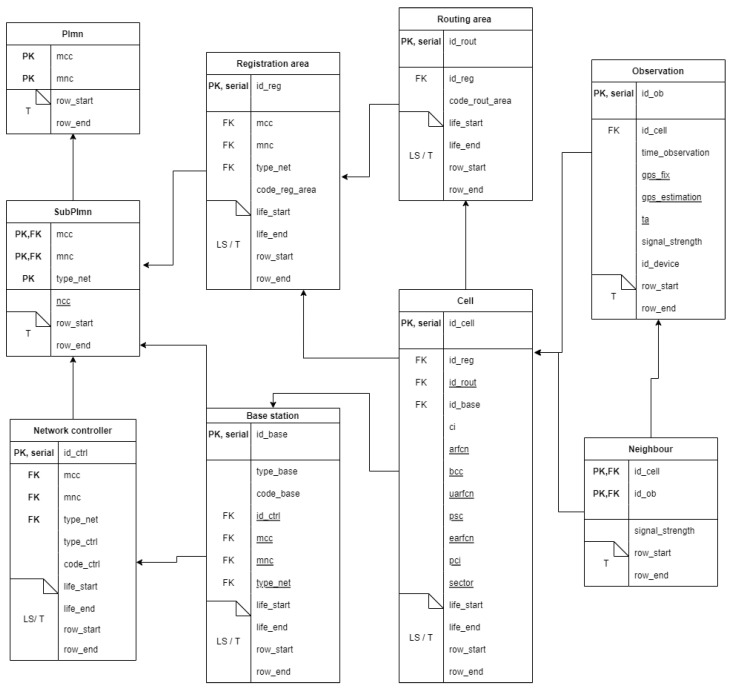
Relational logical model. Underlined attributes can take NULL values. Attributes *life_start* and *life_end* (resp., *row_start* and *row_end*) represent the extremes of the valid time interval (resp., transaction time). Primary key and foreign key attributes are denoted by PK and FK, respectively. The keyword ‘serial’ highlights the surrogate identifiers.

At this point, the restructured cellular network conceptual schema of Figure 7 can be translated into a relational one using the standard mapping rules [36], and then extended with the support for spatial and temporal features [34]. The resulting relational logical schema is given in Figure 8. Here, underlined attributes can take on NULL values; attributes *life_start* and *life_end* represent the extremes of the valid time interval (LS); and attributes *row_start* and *row_end* represent the extremes of the transaction time interval (T). Primary key and foreign key attributes are denoted with PK and FK, respectively. The keyword ‘serial’ highlights the surrogate identifiers.

#### 3.7.2. Notes on the Physical Implementation

The logical schema was implemented in a PostgreSQL 13.3 database instance. Since PostgreSQL does not provide a comprehensive native support for spatial features, we relied on a PostGIS extension for that. Similarly, for the temporal features we relied on the third-party temporal extension Periods (https://github.com/xocolatl/periods, accessed on 30 October 2022) which provides, by means of history tables, bi-temporality constructs (i.e., it handles both transaction and valid time) and a large number of predicates for temporal attributes, all in compliance with the behavior defined in the standard SQL:2016.

There are some important technical notes: The database includes a set of SQL triggers to deal with the automatic population of the majority of the tables, starting with recorded observations. For instance, when a new observation is inserted in the table “Observation”, the corresponding cell is automatically inserted in the table cell (if the latter is not already present in the database). All other involved entities are treated in a similar way, with the result of the configurations of cellular networks being entirely reconstructed, starting from the inserted fingerprints. Specific functions have been developed to obtain the spatial coverage of entities (e.g., PLMN, SubPLMN, and cell) and to merge two instances involved in a renaming episode (updating the parameters of the cell that has been renamed with the new values). The idea is that the system should run this last function periodically, in order to foster network data consistency.

Finally, as the overall size of the database can grow to be quite large, adequate index structures have been defined to speed up the most frequent queries expected to be run against the database (see, for example, Section 5 for some use cases). In particular, considering coverage calculation, indices have been defined over the tables of the entities that belonged to a spatial aggregation (e.g., this is the case of observation’s attribute *id_cell*).

## 4. Cellular Network Reconstruction and Validation

The implemented database allows for the crowdsourced collection of a large amount of information from several sources to derive deep knowledge about the global cellular network, irrespective of the specific technology involved.

In our scenario, where radio cellular signals are exploited for estimating the position of a device, the measurements acquired by the latter are the only source of information that can be used to extract knowledge about the network. Since information may be easily incomplete or affected by errors, we have been forced to relax some logical constraints (e.g., not null) to deal with missing data. Subsequent analyses have also been designed by taking such issues into account.

As already mentioned, several projects have started worldwide with the objective of collecting spatio-temporal information about cellular networks in a collaborative form. Among the public ones, we considered *OpenCellID*, due to its size and popularity. In addition, we also obtained a proprietary anonymized dataset thanks to the contribution of the company *u-blox*. Both datasets are collections of measurements, i.e., recordings pertaining to the detection of a serving or neighbor cell (that, in our schema, correspond to table Observation and table Neighbour, respectively).

In the following, after a short introduction of the two datasets (Section 4.1), we move on to the population of the database, focusing on two different aspects: the validation of the measurements (Section 4.2) and the automatic generation of network information (Section 4.3).

### 4.1. Considered Datasets

**Figure 9:**
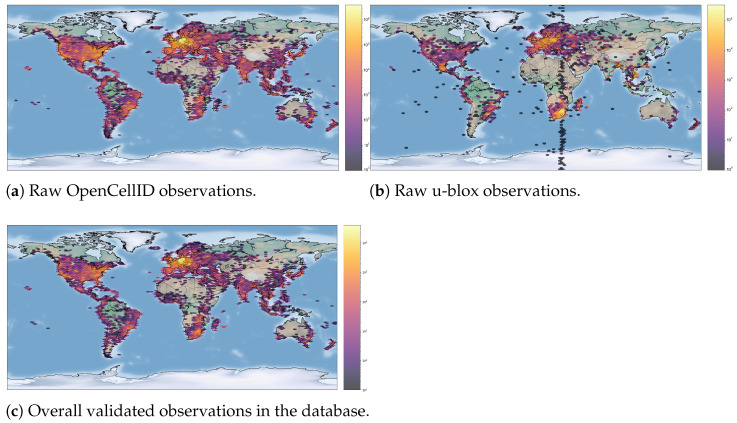
Observations spatially grouped by hexagons. Color = number of observations per hexagon.

*OpenCellID.* It is a collaborative community project that collects measurements and cell towers’ data by means of an API and a ready-to-use mobile phone application. In spring 2017, the project was acquired by Unwired Labs, a geolocation service provider enterprise. This step changed privacy policies and also the kind of published data. Regardless, we worked on a dataset downloaded from the site project in April 2017. The data are in *csv* tabular format, and each measurement is characterized by the following attributes: *mcc*, *net*, *area*, *cell*, *lon*, *lat*, *signal*, *measured*, *created*, *rating*, *speed*, *direction*, *radio*, *ta*, *rnc*, *cid*, *psc*, *tac*, *pci*, *sid*, *nid*, and *bid*. Clearly, depending on the specific technology, some features may not be available, and their meanings may even be different. The problem of missing data is also exacerbated by the fact that the hetereogeneous devices which contributed to the dataset may have provided different subsets of information. The original dataset includes 42,952,377 measurements based on three different cellular technologies: GSM (26,896,809), UMTS (6,195,903), and LTE (9,859,665). The dataset covers the entire world, as can be seen in Figure 9a, where different densities in different areas can also be appreciated. OpenCellID dataset makes no distinction between serving and neighbor cells; thus, all measurements are considered as to be distinct, and all entries are serving cells.
*u-blox.* For further testing the generality of our proposal, we extended the OpenCellID data with a proprietary dataset gathered by the company u-blox. For privacy reasons, we have obtained only information about the cellular networks, and not any details about devices and users. The dataset was assembled by parsing raw measurements logs. Each measurement contains a set of cells that are explicitly identified as serving and neighbors. Differently from OpenCellID, this dataset includes only GSM and UMTS cell information. GSM serving cells might contain a TA value, whereas UMTS neighbor cells do not usually have the logical parameters but only the physical ones. Measurements include the GNSS position with the time to first fix (TTFF), and the number of detected satellites. Overall, the dataset includes 12,492,545 measurements, partitioned into GSM (11,998,811) and UMTS (493,734). Figure 9b shows that, with respect to the OpenCellID dataset, u-blox data have lower worldwide coverage, although some areas are more densely sampled—for instance, South Africa. A high density of observations is also present in Europe, which, as we shall see, is useful for comparison and integration with data from OpenCellID.

### 4.2. Measurements Validation

When inserting new measurements in the cellular network database, it must be taken into account that each possible data source may use specific units or formats for storing the captured parameters; since our main goal is dealing with heterogeneous data sources, we adopted specific conventions that have to be followed in the database. As an example, for the signal strength there are different measurement units: relative indexes such as received signal strength indicator (RSSI) [28], or instead, row values expressed in absolute numbers representing power in decibels relative to a milliwatt (dBm). We chose to rely on the latter and convert the measurements accordingly.

**Figure 10:**
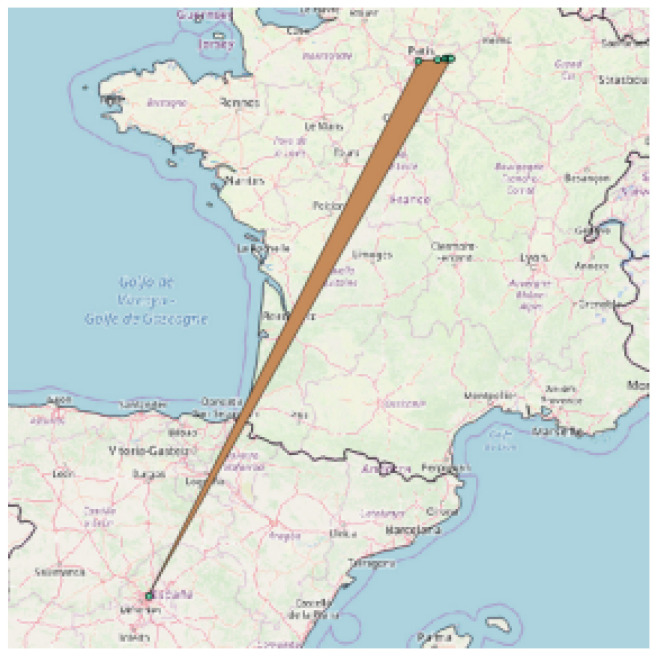
Oversized cell caused by an inter-country faulty observation.

In addition, as measurements come with errors (see, e.g., the vertical line of observations depicted in Figure 9b), it is necessary to take all possible actions to guarantee the quality of the data ultimately stored in the database. Thus, an appropriate a set of constraints has been defined to validate every new measurement before its actual insertion. A common case is when an observation contains one or more values out of range, which could be a valid reason to entirely discard it. Domain ranges for each attribute and technology are summarized in Table 2. Other anomalies that can be easily detected are the cases where some attributes are incorrectly set; for instance, where rnc=ci (in OpenCellID, also =cell) or where some identifier have NULL values. Furthermore, the GNSS position allows us to add more complex constraints to the incoming data. For example, a measurement characterized by the presence of GNSS data is kept only if it has at least three visible satellites, avoiding poor readings that could reduce the overall quality of the database. Another interesting check is to verify whether the GNSS location of an observation is contained within the borders of the country corresponding to the associated mcc. Specifically, country borders were considered with a 20 km buffer to retain cases where the radio signal can be received barely outside of them. In this regard, consider Figure 10, reporting a situation in which this last check is not carried out, and as a result, an observation referring to the French mcc is considered valid although being very far from its boundaries, generating exaggerated coverage for the related cell (orange polygon).

Although in this work we applied the previously described conditions only to serving cell observations, a subset of them may also be applied to neighbor cells.

### 4.3. Automatic Generation of the Network Database

Following the hierarchical granularity structure of cellular networks, from the smallest component, that is, the cell, to the biggest one, the PLMN, the network schema was automatically populated, inserting or updating data as necessary. For example, when a new valid observation (according to the criteria defined in Section 4.2) is inserted, the corresponding (serving) cell has to be considered. If the latter is not already present in the table cell, it has to be added, and such an operation is followed by the insertion, or the update, of the overlying network components, such as the corresponding registration area.

To simulate the continual, crowdsourced arrival of cellular observations, we populated the database as follows. The instances inside both datasets are characterized by a value that denotes their time of capture (*measured* in the OpenCellID dataset; *TTFF* in the u-blox dataset). Thus, we first combined the two datasets, sorting the data by such temporal annotations. Then, we inserted the observations one by one in the database, applying the filters defined in Section 4.2 and setting the lower end of the transaction time interval value (i.e., *row_start*) of each instance equal to the time at which it was collected. This simulated and incremental population of the database is useful for evaluation purposes, as shown in Section 5.

After the database population phase, it is worth assessing the result of the online filtering process that we employed. The conditions applied to the original datasets led to the results reported in Table 3. Starting from the original datasets, after the filtering phase, there were 52,050,495 measurements left (45,179,811 serving cells and 6,870,684 neighbors) to reconstruct the network. Only 93.88% of original observations were maintained: 99.80% from the OpenCellID dataset and 73.52% from the u-blox dataset. While for the OpenCellID dataset we do not know if some kind of filtering was already applied, the u-blox dataset was obtained directly from unfiltered raw measurements logs, and this explains its higher amount of discarded observations. Figure 9c depicts the spatial distribution of all the validated instances that have been inserted in the database.

The composition of the overall reconstructed network arising from the merging of both datasets is summarized in Table 4. As shown, there are almost 1000 PLMN, organized in an average of 800 subPLMN for each technology, each one with approximately 45,000 registration areas for each technology; and a total of 5,794,700 distinct cells (1,553,523 for GSM, 2,001,145 for UMTS, and 2,240,032 for LTE). As for the geometries associated with the different elements and calculated using single observations, they should intuitively be characterized by a polygon. However, in several cases, they are points or lines, for instance, because a cell is recognized only by one or two observations. The numbers of constructed polygons are also reported within brackets in Table 4.

**Figure 11:**
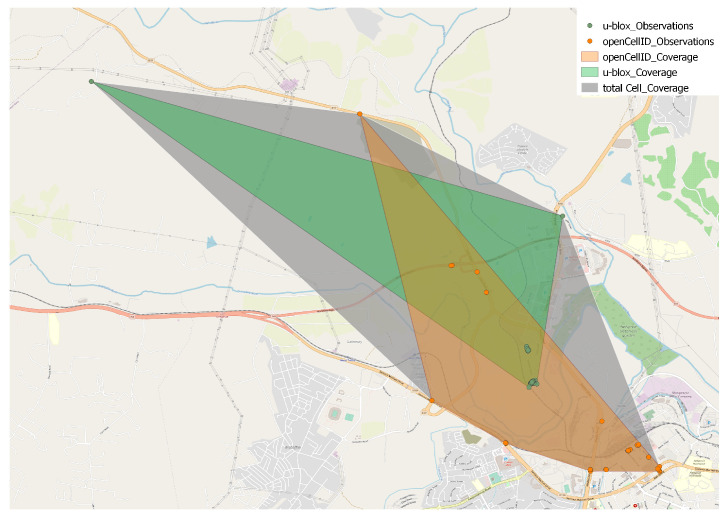
Integration of different sources: a cell reconstructed from 37 observations of the OpenCellID dataset and 87 observations of the u-blox dataset.

The last thing to consider about the two datasets is their temporal extension. OpenCellID includes observations from 2014-01-01 (02:02:44) to 2017-03-17 (06:34:24), whereas the u-blox dataset includes observations from 2016-06-23 (23:21:20) to 2016-06-30 (23:00:57). Note that the latter is a much shorter period than the former, and there is a clear overlap. The overlapping period is very relevant because it allows us to perform analyses and verification concerning the data integration process. As a matter of fact, we were indeed able to easily recognize 63,839 cells appearing in both datasets. An example is shown in Figure 11. The cell identified by type_net=‘GSM’, mcc=655, mnc=2, code_area=1011, ci= 10,503 (grey polygon) was reconstructed using 37 observations of OpenCellID and 87 observations of the u-blox dataset (the orange and green polygons, respectively).

### 4.4. Continuous and Periodic Validation

The previously mentioned filtering operations applied during the database population phase can be referred to as “continuous validity checks”, to underline the fact that they were launched for each new measurement, in contrast with “periodic validity checks”, which are instead run only at regular intervals, due to their semantic nature and computational complexity. An example of a periodic check is as follows: if a cell has enough associated observations, it can be determined whether the (possibly estimated) locations of its latest observations (that is, those entered after the last launch of this periodic check) are consistent with the coverage of the cell—i.e., if the geometric distances of the locations with respect to the previously known cell extension are plausible (e.g., not too large). Finally, the detection of cell renaming phenomena has also been implemented as a periodic routine: briefly, on the basis of [15], we verified whether several (spatio-temporal) conditions characterizing the renaming phenomenon are satisfied. Ideally, periodic checks should complement and be run in parallel with continuous ones.

## 5. Cellular Network Analysis

In the previous section, we showed how the database is capable of supporting several cellular network-related operations, with a focus on integrity checks. Here, we present some other relevant use cases that are made possible by the developed system, concentrating on analysis tasks. Clearly, most of the described analyses can also be performed using raw data, but in that case, the procedures are much more complex and computationally intensive than using our structured model. Overall, all the analyses in this work demonstrate how effectively the proposed model can deliver deep knowledge of the network, which would be very difficult to achieve based on the original datasets separately.

### 5.1. Basic Analyses

**Figure 12:**
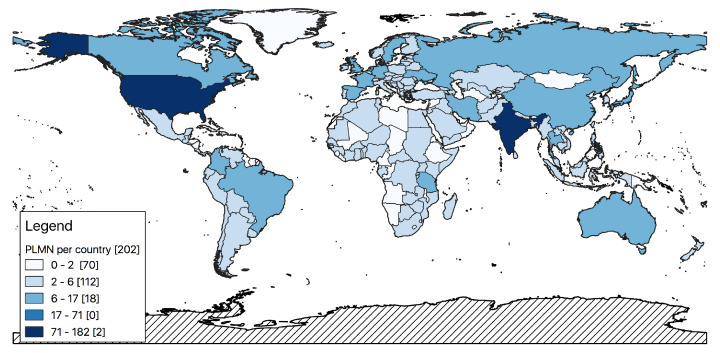
Number of PLMNs per country.

A number of straightforward analyses to extrapolate statistics concerning the cellular network and its configuration can be carried out, involving different components. For instance, suppose that we want to obtain the number of PLMNs available in each country. Since each *mcc* can be related to one or more country boundaries, and given the fact that a country may have several associated *mcc*s, the solution is to group PLMNs (couples of *mcc, mnc*) by the corresponding country *iso* codes to obtain a list of PLMN for each country. Starting from the above grouping, a straightforward representation is the choroplet map where each country is colored from light to dark following the number of PLMN operating in that country, as shown in Figure 12. In this map, dark colored countries have higher numbers of PLMNs according to the breaks described in the legend. Note that for this analysis we used all recognized PLMNs, independently from the associated geometry.

**Figure 13:**
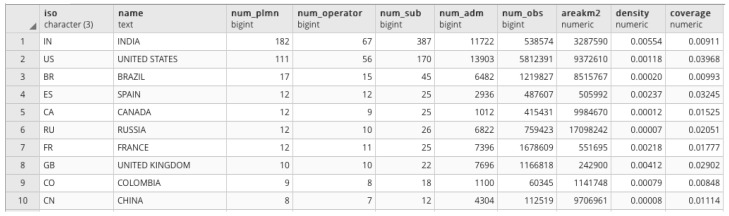
Top ten: PLMNs per country.

One might be interested in finding the 10 countries with the highest number of PLMN operators, leading to Figure 13. The specific legend breaks have been chosen because, as is visible in Figure 13, India and the Unites States have very large numbers of PLMNs (182 and 111, respectively) compared to other countries, which have at most 17 PLMNs. As for the density of the PLMNs (column *density*), by dividing the number of the PLMNs over the areas of each country, we can observe immediately that, except for very small countries (e.g., Monaco, Gibraltar, or Macau), most have a density of under 0.0089 PLMN per Km${}^{2}$ (more than 50%).

### 5.2. Spatial Analyses

**Figure 14:**
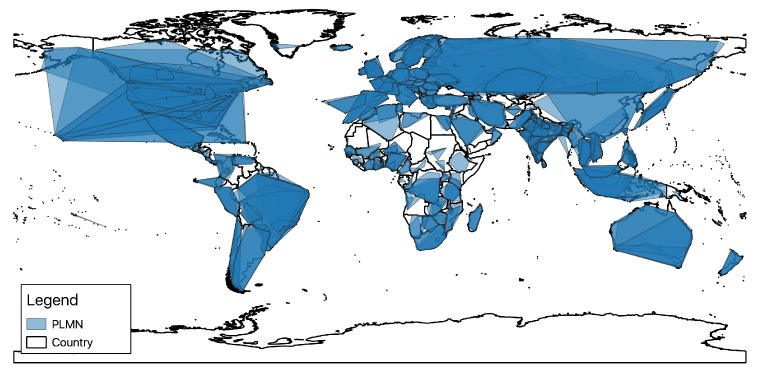
A general overview of PLMN coverage.

As an immediate byproduct, the developed database allows us to instantly obtain a visual representation of network coverage across the entire globe. For instance, as shown in Figure 14, we can directly display the derived coverage area of each PLMN. Obviously, the depicted polygons are the convex hulls built from all the validated observations belonging to each specific PLMN. For this reason, geographical areas not covered by any polygons do not necessarily correspond to zones with no radio coverage at all, but are probably characterized by a number of points too small to build a polygon.

At this point, thanks to the structuring of our model, other information can be immediately retrieved at each level of the administrative organization of the cellular network and with respect to different technologies. For instance, let us consider Germany, which is the country with the maximum number of observations (21,713,580) and the second according to their density (after Singapore) in our database; it is the second country by number of cells (560,803, after US) and the twelfth for their density. In the following, we show how different levels of the network architecture hierarchy provide different kinds of information regarding the chosen area.

**Figure 15:**
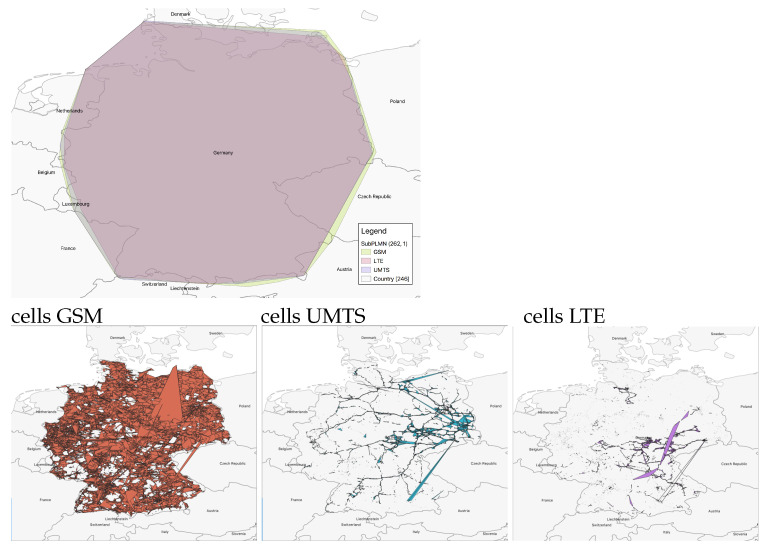
Coverage of different technologies by the same PLMN: mcc = 262 (Germany) and mnc = 1.

**Figure 16:**
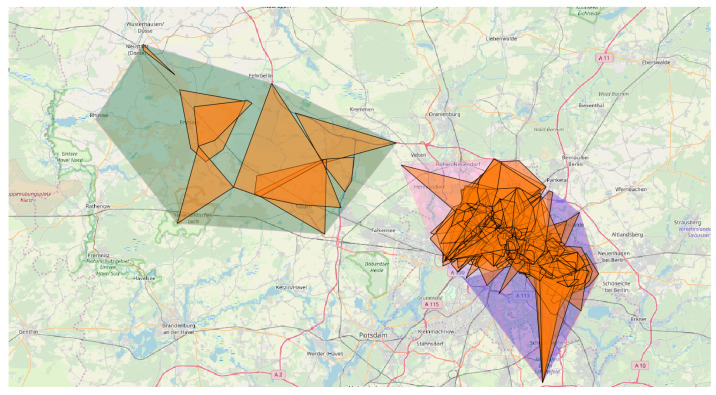
Administrative areas in the Berlin area: urban (violet and pink polygons) and rural (green polygon) area. Orange polygons represent cell coverage.

**Figure 17:**
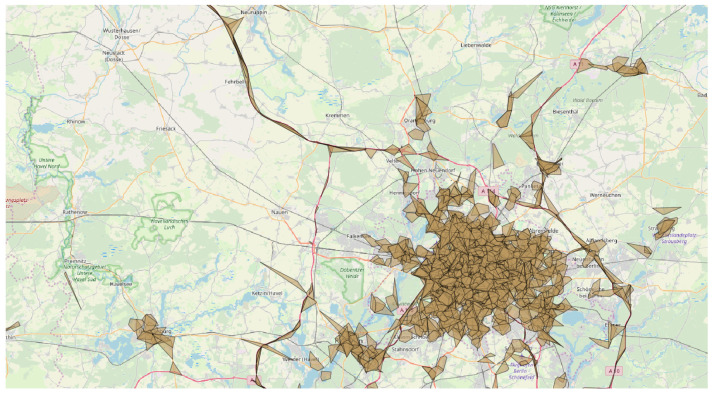
Distribution of microcells in the Berlin area.

*subPLMNs*. This is the case where one wants to inspect the coverage for a given area with respect to a single PLMN and/or a specific technology. Analyses like these may, e.g., point out which PLMN has the best coverage with respect to a specific cellular technology. While comparing different technologies and working at the level of subPLMN, it is immediately clear that around 50% of PLMNs (combination of mcc and mnc) were detected to have all three considered technologies (457 out of 996). Note that 302 had two technologies, and only 237 had only one. As an example, Figure 15 (top part) shows the coverages associated with the three different technologies considered in this work for a German PLMN (mcc = 262, mnc = 1). Although their areas may look very similar, if we calculate the bounding boxes at the cell level (still grouping them by subPLMN), we obtain a very different picture, as shown in Figure 15 (bottom part). This remarks on the usefulness of structuring the information at different granularity levels, modeled within a flexible hierarchy.
*Administrative areas*. Proceeding down the hierarchy of the network, we find the logical grouping of cells represented by administrative areas. These play a major role in localization at a coarser granularity, as described in [37,38,39]. Some works [37,40,41] point out that the density of the cells within an area is likely useful to distinguish between urban and rural environments. In such a context, administrative areas are a simple way to sample the territory for computing the density and check whether it is a rural or an urban area: our model makes it easy to compute the density of cells following the administrative partitioning. As an example, considering the GSM network with mcc=262 and mnc=3, Figure 16 shows some administrative areas close to the city of Berlin, each one being characterized by at least 9000 related observations. The violet and pink polygons correspond, respectively, to administrative areas identified by lac 21493 and 20473 covering two urban areas, and the green polygon on the left is the administrative area with lac 25503—in essence a rural area. It can be easily seen that the pink and violet polygons are characterized by a density of cells far higher than the green one.
*Cells*. As described in [40] cells can be split into two categories: macrocells and microcells. Macrocells are usually related to a higher transmitting power, leading to a larger coverage area, whereas microcells are smaller and low-powered. The latter are typically used as support for extending networks’ capacity for specific areas, such as malls or crowded places. Urban areas are likely to contain more microcells than rural areas [40]. Finding an optimal strategy to discriminate microcells against macrocells is out of the scope of this paper; anyway, by restricting our attention to cells characterized by an area of at most 5 km${}^{2}$ and containing more than 30 observations, we can get an idea about this difference, as reported in Figure 17.

### 5.3. Temporal Analyses

In general, a set of observations represents a given state of the network. As for their sampling, neither spatial nor temporal regularities are guaranteed. Thus, no assumption can be made about the state of the network between two measurements involving the same cell, especially if they are not close in time. As an example, it is not possible to establish whether a cell was not visible because it was not operating or simply because no device had made observations in its coverage area. In the following, under the simplifying assumption that the latter hypothesis holds, we outline some useful temporal analyses.

**Figure 18:**
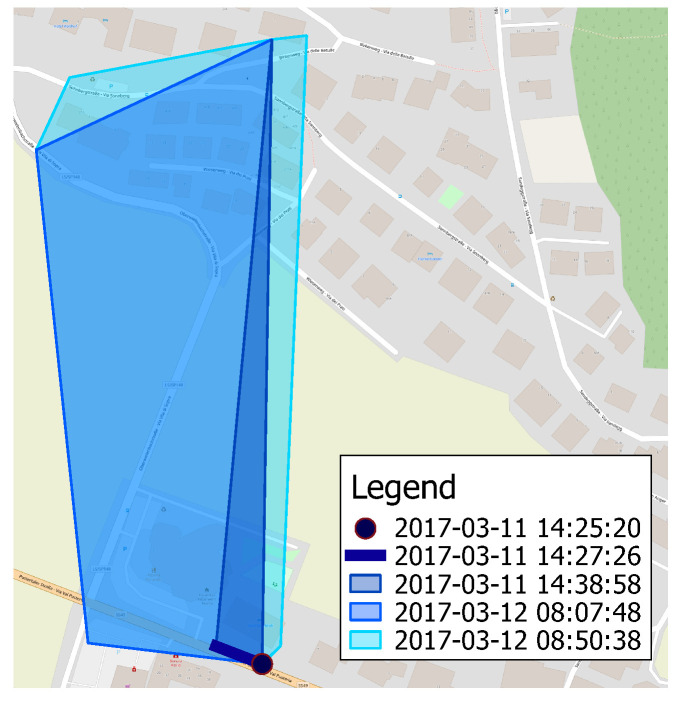
Evolution of the coverage of a cell as new measurements are added to the database over time. A brighter color denotes a more recent state.

**Figure 19:**
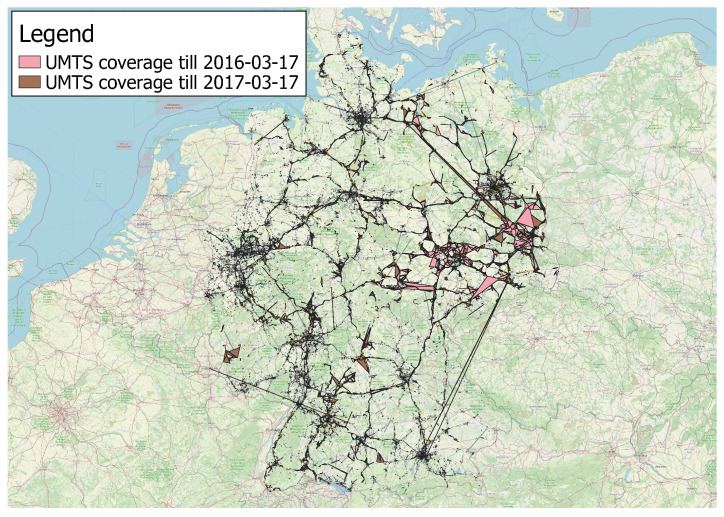
Temporal evolution of the UMTS coverage in Germany: measurements were obtained from 2015 to 03-17 to 2016-03-17 (pink color) or 2017-03-17 (brown color). Only serving cells are considered.

**Figure 20:**
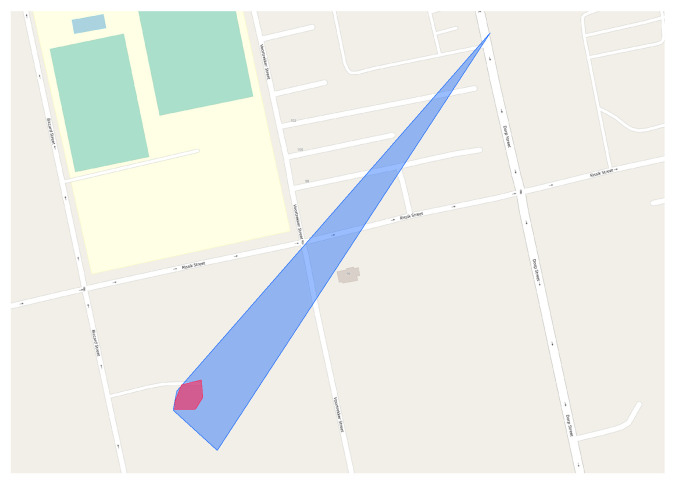
Backtracking a cell-renaming operation. The two original cells are shown in red and blue.

*Cell coverage evolution*. The presence of both transaction time and valid time dimensions allows us to easily track the evolution of the coverage of a cell over time. The idea is to exploit the transaction time of the involved instances to easily rollback the state of a cell. Figure 18 illustrates the coverage of a cell in the province of Bolzano (Italy) as it changes over time. Specifically, we report the resulting shape as new observations for such a cell are made. At the beginning, the coverage is just a single point, as only one observation is detected in such a cell. Then, the area progressively grows till it reaches the extension of the bright cyan polygon on 2017-03-12 at 08:50:38, i.e., when the last observation of the cell is added to the database. It is worth pointing out how the overall knowledge about the cell dramatically changed over a very short time interval.
*Cellular technology coverage evolution.* Let us now turn to another practical scenario where the temporality of the data shows its usefulness: based on the valid time recorded for each instance, we can easily inspect the evolution of the coverage of the UMTS network in Germany at two different time points (2016-03-17 and 2017-03-17), as shown in Figure 19. Note how the coverage increased over time. Similar analyses can extract the evolution of the coverage of several mobile operators considering different technologies. In turn, those data may allow one to detect deficiencies in an operator’s network, to compare competitors’ coverages, and to build machine learning models able of predicting their future extension.
*Cell renaming.* Here we present the case of an actual cell that has been obtained after a renaming operation, detected thanks to the developed system. In such a case, it may still be useful to investigate how the network arrangement was before the renaming operation. Figure 20 depicts the coverage of a cell in the city of Polokwane (South Africa) as currently stored in the database. Thanks again to the support of valid and transaction times offered by the system, we can easily roll back the renaming operation, showing the previous situation where two cells are visible (red and blue polygons).

## 6. Conclusions

In this work, we conducted a systematic study on how it is possible to reconstruct and maintain information about the infrastructure of cellular networks by making use of crowdsourced data sensed by mobile devices. Even though each generation of cellular networks is based on standard specifications, the task is far from being trivial, as each mobile operator adopts its own organization and makes some changes, usually not known by the external people. Nevertheless, comprehensive and reliable knowledge of the network is of primary importance in many fingerprint-based outdoor positioning tasks, ranging from navigation to contact tracing and emergency management.

The gained knowledge was formally encoded by means of a conceptual database schema that is flexible enough to deal with several kinds of network technologies (and easily extendable to others) and able to accommodate crowdsourced measurements. This schema was then translated into a relational one, and subsequently implemented in a spatio-temporal database running on a PostgreSQL DBMS. The database was populated using two (distinct, although temporally overlapping) datasets, one public, obtained from the OpenCellID repository, and the other private, gathered from the company u-blox. As a result, the overall network information was reconstructed. Based on the collected data, we then showed how the system is capable of supporting several network-related tasks. Most importantly, it allows one to maintain an accurate and up-to-date representation of the network infrastructure, through the detection of inconsistent measurements coming from mobile devices, e.g., due to the violation of spatio-temporal constraints on their collection, and cell renaming phenomena. In addition, a selection of exemplary network analyses have been presented, ranging from basic ones to more complex spatial and temporal use cases. Overall, the proposed system poses as a solid basis to foster all kinds of tasks based on outdoor positioning and cellular network analysis.

As for future work, given that our database structure is highly flexible and modular with respect to the considered network technologies, we plan to extend the spatio-temporal database with the support for 5G. In addition, to further promote network analyses, other datasets will be integrated within the database.

## Figures and Tables

**Table 1:** Acronyms related to the specific network technologies used throughout the paper.

Acronym	Technology	Description	Acronym	Technology	Description
ARFCN	GSM	Absolute Radio Frequency Channel Number	PCI	4G	Physical Cell Identifier
BCC	GSM	Base station Color Code	PCU	GSM	Packet Control Unit
BSC	GSM	Base Station Controller	PLMN	All	Public Land Mobile Network
BSIC	GSM	Base Station Id Code	PSC	UMTS	Primary Scrambling Code
BTS	GSM	Base Transceiver System	RA	GSM, UMTS	Routing Area
CGI	GSM, UMTS	Cell Global Identifier	RAC	GSM	Routing Area Code
CI	GSM, UMTS	Cell Identifier	RAI	GSM	Routing Area Identifier
EARFCN	LTE	Evolved ARFCN	RNC	UMTS	Radio Network Controller
ECI	LTE	E-UTRAN Cell Identifier	RSCP	UMTS	Received Signal Code Power
LA	GSM, UMTS	Location Area	RSRP	LTE	Reference Signal Received Power
LAC	GSM, UMTS	Location Area Code	RXLEV	GSM	Receiving Level
LAI	GSM, UMTS	Local Area Identifier	TA	LTE	Tracking Area
MCC	All	Mobile Country Code	TAC	LTE	Tracking Area Code
MNC	All	Mobile Network Code	TAI	LTE	Tracking Area Identifier
NCC	GSM	Network Control Code	UARCFN	UMTS	UTRA ARFCN

**Table 2:** Domain constraints on measurement’s parameters.

	GSM	UMTS	LTE
**MCC**	0–999 (3 digits)		
**MNC**	0–999 (3 digits)		
**LAC/TAC**	0–65,535		
**CI/eCI**	0–65,535		0–268,435,455
**RNC**	-	0–4095	-
**(U/E)ARFCN**	0–1023	0–16,383	0–65,535
**BSIC/PSC/PCI**	0–63	0–511	0–503
**TA**	0–219	-	0–1282

**Table 3:** Measurements (1 = OpenCellID, 2 = u-blox).

Dataset	Tech.	Observations	Valid Observations	%	Neighbors	Valid Neighbors	%
1	GSM	26,896,809	26,840,087	99.79	0	0	0
1	UMTS	6,195,903	6,177,024	99.70	0	0	0
1	LTE	9,859,665	9,848,455	99.89	0	0	0
2	GSM	2,522,483	2,258,522	89.54	9,476,328	6,768,116	71.42
2	UMTS	62,318	55,723	89.42	431,416	102,568	23.77
1		42,952,377	42,865,566	99.80	0	0	0
2		2,584,801	2,314,245	89.53	9,907,744	6,870,684	69.34
1&2		45,537,178	45,179,811	99.21	9,907,744	6,870,684	69.34

**Table 4:** Overall network summary: total number of elements in our database (polygons in brackets).

	CELL	REGISTRATION AREA	SubPLMN	PLMN
GSM	1,553,523 (1,553,523)	45,970 (35,157)	811 (743)	
UMTS	2,001,145 (693,622)	43,147 (29,139)	794 (746)	993 (925)
LTE	2,240,032 (864,673)	48,098 (40,787)	607 (559)	
	5,794,700 (3,111,818)	137,215 (105,083)	2212 (2048)	

**Figure A1:**
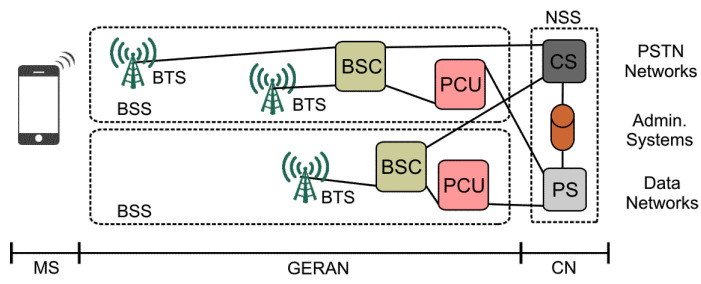
The architecture of GSM/GPRS/EDGE cellular networks.

**Figure A2:**
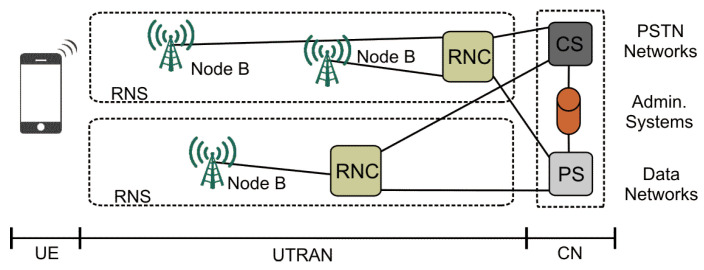
The architecture of UMTS cellular networks.

**Figure A3:**
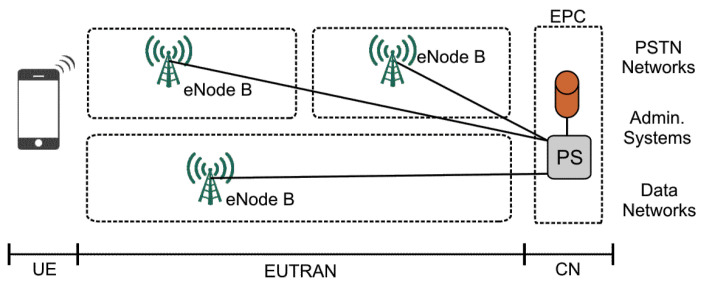
The architecture of LTE cellular networks.

## Data Availability

Not applicable.

## Appendix A. Cellular Network Technologies

Here we describe, from the data-modeling perspective, the technical details pertaining to different cellular network technologies, including GSM, UMTS, and LTE. Once more, observe that the standard network specifications have been proven useful to only provide us with general guidance, though the definitive information could be achieved by thoroughly analyzing the data received by mobile devices.

### Appendix A.1. 2G Global System for Mobile Communications (GSM)

In the early 90s, the Global System for Mobile Communications (GSM), also referred to as the second generation (2G) of cellular networks, significantly changed the way people communicated, as proven by the billions of subscribers. GSM was first designed and developed in the 80s for voice and data communication using circuit switching only, and then extended with packet-switched functionalities to better fit discontinuous data mobile applications (such as Internet browsing). This was done by introducing the General Packet Radio Service (GPRS), and then the improved Enhanced Data rates for GSM Evolution (EDGE), which exhibited faster transfer rates.

#### Appendix A.1.1. Architecture

In GSM cellular networks, devices are usually called mobile stations (MSs), and they allow people to communicate with the GSM/GPRS/EDGE radio access network (GERAN or 2G RAN), as shown in Figure A1.

The GERAN is organized into base station subsystems (BSSs). Each subsystem was originally composed by two architectural elements: the base transceiver system, also referred to as BTS (BTS is equivalent to a BS, or base station, in 3G and 4G. For this reason, a BS is often used also in 2G for BTS), which includes several components, such as transmitters, receivers, and other equipment for wireless air-interface operations; and the base station controller (BSC), which is basically a switch in charge of frequency management among the BTSs belonging to the same BSS. Each BSC can control from one to hundreds of BTSs. Later, to support GPRS packet switching, an additional component called the packet control unit (PCU) was added [28]. It controls the packet traffic, and its most common position is right after the BSC, as shown in Figure A1.

At the highest level, the network and switching subsystem (NSS) supports the core network services for both circuit-switching (CS) and packet-switching (PS) connections.

#### Appendix A.1.2. Cell Global Identifiers

In order to uniquely identify a cell at a given instant, 2G networks make use of four parameters that differentiate each cell from all other cells in the world: the MCC, the MNC, the location area code (LAC), and the cell identifier (CI). The first two parameters identify the PLMN. The other two are defined as follows:

- The LAC is a fixed-length code (16 bits/4 digits) that characterizes a location area (LA) in a GSM network;
- The CI is used to uniquely identify a cell in a given LAC (this means that the same value for CI can occur in different LACs). It consists of 16 bits and can thus assume 65,535 different values. In the early years of the GSM technology, due to the small number of cells in a network, the CI was unique for all the LACs.

The concatenation of MCC, MNC, and LAC is usually called a local area identifier (LAI).

In GPRS networks, besides the location area, there exists a routing area (RA), which is related to the data packet traffic across the network. Each RA is included in an LA; in general, an LA can include one or more RA. Each RA is identified by a routing area identifier (RAI), which is the concatenation of the LAI with a fixed-length code (8 bits/2 digits) called the routing area code (RAC).

Finally, it is worth pointing out that there seems to be an interesting connection between the components of a GSM PLMN and the CIs. In some cases (see, for instance, http://www.erlang.com/forum/erlang/thread.htx?thread=686 (accessed on 1 February 2022)), indeed, the identifiers of these components can be extracted from the CIs of the cells, as the CI of a cell, say, xXYYZ, consists of the BSC number xX, the BTS number YY relative to the specific BSC, and cell/sector Z relative to the specific BTS (0 in omnidirectional BTSs; from 1 to 3 in the case of sectoral ones).

#### Appendix A.1.3. Physical-Layer Cell Identifiers

In 2G GSM, cells are identified at the physical layer via the base station ID code (BSIC), transmitted over the synchronization channel (SCH) about 25 times per second, giving the opportunity to quickly gather enough information to discriminate the received cells. It consists of 6 bits, which are usually structured as follows (such an organization is not mandatory):

- The three most significant bits are the network color code (NCC), which can be used to distinguish one operator from the other, as in a country, a distinct NCC is assigned to each operator;
- The latter three bits are the base station color code (BCC), which can be used to discriminate among the cells associated with the same network operator.

In principle, neighboring cells should have a different BCCs, but, since there are only $2^{3}$ distinct values available, there is often the need to reuse codes. In order to uniquely identify the cells, one can pair the BSIC with the absolute radio frequency channel number (ARFCN). The ARFCN is a short description for the radio carrier resources (a pair of downlink/uplink), and its value may vary from 0 to 1023 (not all values are used). The same pair (BSIC, ARFCN) cannot be reused within the same region of the network.

### Appendix A.2. 3G Universal Mobile Telecommunications System (UMTS)

The Universal Mobile Telecommunications System (UMTS), first released in 1999, is the third generation (3G) of cellular systems and represents the natural evolution of the second generation (2G) systems. It combines voice and data services, and it supports interworking with existing networks.

#### Appendix A.2.1. Architecture

As shown in Figure A2, in UMTS cellular networks, devices are usually referred to as user equipment (UEs). From 3G onwards, the mobile equipment (ME) is usually endowed with a universal SIM (USIM), an advanced SIM which is able to handle several mini applications and has much more memory.

The UMTS terrestrial radio access network (UTRAN) is partitioned into several radio network subsystems (RNSs). Its organization is similar to that of GSM networks. Each RNS consists of a set of radio elements, called base stations (BSs) or simply NodeBs, and their corresponding controlling elements, called radio network controllers (RNCs).

The latter part of the architecture, called the core network (CN), covers all the network elements needed for switching and subscriber control.

#### Appendix A.2.2. Cell Global Identifiers

In UMTS, cells are grouped into distinct administrative areas which depend on the specific service.

As for the registration service, cells are assembled into location areas (LAs), like in the case of GSM. LAs are identified by a location area identifier (LAI), which pairs the PLMN identifier with the LAC (16 bits).

As for data routing, we refer to routing areas (RAs). In general, an LA consists of one or more RA [42]. Each RA is identified by the routing area identifier (RAI), which pairs the LAI with the 8-bit Routing area code (RAC). LACs and RACs are broadcast on a periodic basis over the BCCH.

As for the administrative organization of the network, as in GSM, UMTS cells are uniquely identified by means of the concatenation of MCC, MNC, LAC, and CI (16 bits) [43]. In addition, each cell can also be uniquely identified with respect to the architectural structure of the network by replacing the LAC with the 12-bit radio network controller identifier (RNC-ID), which identifies the corresponding RNC [29].

#### Appendix A.2.3. Physical-Layer Cell Identifiers

Similarly to GSM, UMTS provides two physical parameters to locally identify a cell, namely:

- The UTRA absolute radio frequency channel number (UARFCN), which is the radio carrier identifier, as in GSM; it is equal to five times the carrier frequency in MHz and ranges from 0 to 16,383 (14 bits);
- The primary scrambling code (PSC), which is the first part of the synchronization channel (SCH), a downlink signal used for cell search; it ranges from 0 to 511 (9 bits), and it allows one to identify the transmission in each cell.

If the network is properly configured, it is impossible to detect the same pair (UARFCN, PSC) in the same area.

### Appendix A.3. 4G Long-Term Evolution (LTE)

Long-term evolution (LTE) represents the fourth generation (4G) of mobile networks, and it is the successor of UMTS, which it aims to improve on. The standardization of LTE by 3GPP ended on 2008. While GSM and UMTS are based on the circuit-switched model, LTE supports only packet-switched services. It provides seamless Internet Protocol (IP) connectivity between a mobile device and the packet data network (PDN), without any disruption to the end users’ applications during movement.

#### Appendix A.3.1. Architecture

As shown in Figure A3, the organization of the devices that are used to access the network in LTE and the way in which we describe them are the same as in UMTS networks: user equipment (UEs) equipped with USIMs.

Unlike GSM and UMTS, the evolved UMTS terrestrial radio access network (E-UTRAN) that manages the radio communications between the devices and the core network consists of just one component: the evolved base station or eNodeB (eNB). An eNB is essentially a base station that controls the mobile devices in one or (usually) more cell.

The core network, called the evolved packet core (EPC), includes all the elements needed for conveying the user traffic over the network and all the services for session management, security, and administration. Unlike the previous technological solutions, EPC contains only the PS module, as both voice and data services are managed without the need for establishing a circuit connection.

#### Appendix A.3.2. Cell Global Identifiers

Given the reorganization in the RAN level of LTE architecture, differently from GSM and UMTS, all functions are demanded of eNBs. Following the architectural organization of the network, similarly to UMTS, each LTE cell has a global identifier called a EUTRAN cell global identifier (ECGI) that combines the PLMN identifier (MCC + MNC) with:

- eNB-ID, which identifies the eNB responsible for managing the cell;
- cell_ID, which identifies a cell/sector within a specific eNB.

Following the 3GPP standard, the combination of eNB-ID and cell_ID, also known as eCI, is composed of 28 bits: the most significant 20 bits should be the eNB-ID, and the rest should represent the cell_ID. Unfortunately, the network operators are free to partition the 28 bits in any way they wish [29].

Administrative areas in EUTRAN are called tracking areas (TA), and they can include single cells or cover an entire set of cells (as a small town) [29]. The tracking area code (TAC) is a 16-bit code broadcast for each eNB by the System Information Block Type 1 (SIB1) about every 80 milliseconds [44]. In the case when a mobile device UE is in idle mode, a page request is sent all over the eNB belonging to the TA where the UE was last registered. TAC is unique within a PLMN, and therefore, each TA is globally identified by the tracking areas identifier (TAI), defined as MCC + MNC + TAC. It is used to indicate to eNB to which TA the eNB belongs (which cell). When a UE connects to a LTE network, it receives a TAI list indicating areas where it can move without sending a tracking area update request. Finally, note that, from the administrative point of view, eCI, combined with TAI, uniquely identifies a cell.

#### Appendix A.3.3. Physical-Layer Cell Identifiers

LTE networks are designed to employ both single and multiple-frequency cell deployment techniques. For single-frequency deployment, all neighboring cells and sectors use the same channel, and interference is a consequence. In a similar fashion to UMTS, the physical-layer cell identifier in LTE includes two attributes:

- Evolved absolute radio frequency channel number (EARFCN), a 16-bit code that represents the channel number and is bound to the used frequency by a formula;
- Physical cell identifier (PCI), a 9-bit code which is composed of the physical group ID and the physical cell ID. A typical LTE deployment scenario is a three-sector installation on same EARFCN, where each sector has a sequential PCI. The standard defines 168 cell identity groups, each having three identities; thus, there are 168 × 3 = 504 PCI values available.

### Appendix A.4. Summary of Identifiers

The identification of cells, together with the other components, is the most relevant goal in a positioning system based on cellular networks, and a very relevant point in our work. As shown in Table A1, depending on the technologies, different kinds of logical attributes can be collected from an administrative point of view, leading to different cell global identifier constructions (concatenation of italic attributes in Table A1).

**Table A1:** Administrative view of cell identifiers.

Technology	Country	Operator	Administrative Area		Cell
			Voice	Data	
2G GSM	*MCC*	*MNC*	*LAC*	RAC	*CI*
3G UMTS	*MCC*	*MNC*	*LAC*	RAC	*CI*
4G LTE	*MCC*	*MNC*	*TAC*		*eCI*

As already mentioned, the retrieval of cell global identifiers can take up to some milliseconds, and to avoid delays, physical-layer cell identifiers can be used for quick and local identification of the available cells. As shown in Table A2, these identifiers are composed of two parts that depend on the considered technology: the frequency channel number (ARFCN for GSM, UARFCN for UMTS, and EARFCN for LTE) and an additional component, that is, the BSIC (for GSM), PSC (for UMTS), and PCI (for LTE).

**Table A2:** Physical-layer cell identifiers.

Technology	Channel	Physical-Layer
GSM	ARFCN	BSIC (NCC+BCC)
UMTS	UARFCN	PSC
LTE	EARFCN	PCI
